# Transient Electronic Depletion and Lattice Expansion Induced Ultrafast Bandedge Plasmons

**DOI:** 10.1002/advs.201902408

**Published:** 2019-11-27

**Authors:** Xinping Zhang, Meng Wang, Fawei Tang, Huanzhen Zhang, Yulan Fu, Dong Liu, Xiaoyan Song

**Affiliations:** ^1^ Institute of Information Photonics Technology and College of Applied Sciences Beijing University of Technology Beijing 100124 P. R. China; ^2^ College of Materials Science and Engineering Beijing University of Technology Beijing 100124 P. R. China; ^3^ School of Mathematics and Physics Hebei University of Engineering Handan 056038 P. R. China

**Keywords:** lattice expansion, lowering of Fermi level, plasmonic bandedge shift, reduced threshold for interband transitions, transient electronic band depletion

## Abstract

Strong optical excitation of plasmonic nanostructures may induce simultaneous interband and intraband electronic transitions. However, interaction mechanisms between interband, intraband, and plasmon‐band processes have not been thoroughly understood. In particular, optical‐heating‐induced lattice expansion, which definitely leads to shift of the Fermi level, has not been taken into account in plasmonic studies. Here, it is shown that plasmonic bandedge shift is responsible for the optical modulation on the boundary between plasmonic electron oscillation and interband transitions via investigations on gold nanofilms and nanoparticles. Strong optical excitation induces transient depletion of the conduction band just below the Fermi level through intraband transitions, while the subsequent lattice heating induces transient thermal expansion and hence lowers the Fermi level. Both effects reduce the threshold for interband transitions and therefore push the plasmonic bandedge to the red. These discoveries introduce a first correlation between plasmonic response and optical excitation induced thermal expansion of lattices. The revealed Fermi‐level adjustment mechanism allows alignment of electronic levels at the metal–semiconductor interfaces, which applies to all conductive materials and renders reliable physics for the design of plasmonic or optoelectronic devices.

## Graphical abstract

Optical excitation of gold nanostructures induces transient depletion of the conduction band electrons through intraband transitions, and the subsequent lattice heating results in transient thermal expansion. Both mechanisms lower the Fermi level transiently and consequently lower the threshold for interband transitions, pushing the plasmonic bandedge to the red and producing transient absorption on the boundary between interband and plasmon‐band processes.

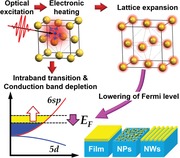

## 1 Introduction

Plasmonic nanostructures or devices have been investigated extensively for potential applications in optical functional elements,^1^, ^2^, ^3^, ^4^, ^5^ sensors,^6^, ^7^, ^8^ photovoltaic^9^, ^10^, ^11^ and light‐emitting diodes,^12^, ^13^, ^14^, ^15^ micro/nanolasers,^16^, ^17^, ^18^, ^19^ optical logic circuits including optical switching devices,^20^, ^21^, ^22^ and other active optoelectronic devices.^23^, ^24^, ^25^ The basic physics may involve optical spectroscopic modulation through optical extinction, light scattering or local field enhancement by surface plasmon resonance, as well as their modifications by strong optical excitation. Surface plasmon is a collective oscillation of free electrons in conductive materials.^26^, ^27^, ^28^ The excitation optical electric field not only drives oscillation of the free electrons, but may also excite inter‐ or intra‐band electronic transitions.^29^, ^30^, ^31^ Energy is then transferred from hot electrons to the phonons, resulting in the heating of the lattices. The spectroscopic response of surface plasmon polaritons (SPP) or localized surface plasmon resonance (LSPR) is strongly modulated with multistage temporal processes.^5^, ^6^, ^7^, ^32^, ^33^, ^34^ Interband excitation has been investigated using ultrafast spectroscopy^35^, ^36^, ^37^ in different metallic nanostructures for the generation of hot electrons, which are important for light‐energy harnessing devices based on plasmon‐mediated optoelectronic processes.

Spectroscopic modulation with multiple features over a broad band has been observed in nearly all of the ultrafast spectroscopic investigation on gold nanostructures in different forms.^31^, ^34^, ^38^ Electron–electron (e–e) and electron–phonon (e–p) scattering have been assigned as the mechanisms for explaining the observations.^39^, ^40^, ^41^ However, these mechanisms are quite general and deep insights for clearer and more straightforward explanations are expected. In particular, the effects on the boundary between electronic bands cannot be simply interpreted by e–e or e–p interactions, which motivates us to disclose the true physics responsible for the electronic and phononic interactions under strong optical excitation at the plasmonic bandedge.

In this work, we discovered that optical excitation of plasmonic nanostructures induce two bandedge‐shift effects: 1) Transient depletion of the conduction band just below the Fermi level through intraband transitions and the subsequent lowering of the threshold for interband transitions. 2) Transient lattice expansion induced lowering of the Fermi level. This is based on the heating of the lattices through energy releasing from the hot electrons to the phonons. These mechanisms explain well the spectroscopic modulations on plasmonic bandedge of the metal nanostructures and are supported solidly by both the experimental and the theoretical evidences. Comparison between the photophysical response of the gold film and gold nanoparticles reveals that the essential physics is not dependent either on the resonance excitation of plasmons or on the forms of the metallic nanostructures. Therefore, similar mechanisms are responsible for the observed effects in both gold films and gold nanoparticles.

## 2 Results and Discussions

### 2.1 Optical Excitation Induced Band‐Structure Modification

**Figure 1:**
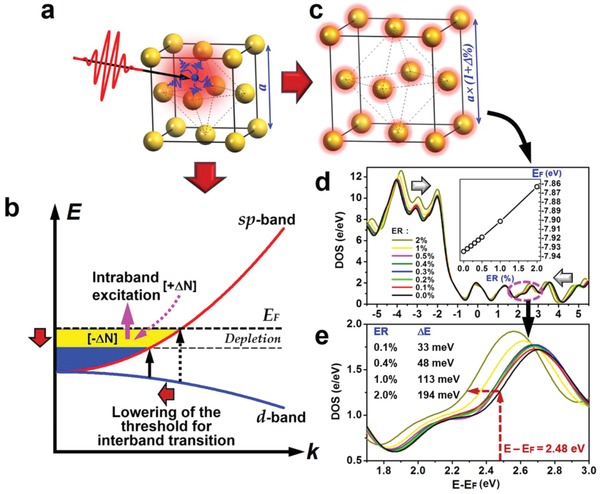
a) Schematic illustration of optical excitation of gold induced heating of the free electrons and subsequent heating of the lattice through electron–phonon interactions. b) Femtosecond optical excitation induced strong intraband transitions and subsequent lowering of the threshold for interband transitions during the relaxation of the hot electrons. c) Linear expansion of the lattice constant *a* by *Δ*% (from *a* to *a* × (1 + *Δ*%)) during the relaxation of the heated lattices after energy transfer from electrons to phonons. d) Calculation of the density of states (DOS) as a function of relative energy (*E* − *E*_F_) with respect to the Fermi level at an expansion ratio (ER) of 0.0%, 0.1%, 0.2%, 0.3%, 0.4%, 0.5%, 1.0%, and 2.0%. The arrows indicate clear narrowing of the bandgap and redshift of the interband transitions. Inset: Variation of Fermi energy with increasing the value of ER. e) An enlarged view of the region enclosed by the dashed circle in (d) for the visible spectral band (1.77 eV < *E − E*_F_ < 3.0 eV).

As generally accepted, optical excitation of gold nanostructures first induced collective oscillation of free electrons, which relaxes through electron–electron interactions within 100 fs.^39^, ^40^, ^41^ Energy will be transferred rapidly from the hot electrons to phonons, leading to the heating of the lattices, which takes a few picoseconds.^39^, ^40^, ^41^ Eventually, all of the energies driving electronic oscillation were released to the lattices and relax as pure phonon processes. **Figure** **1**a describes schematically what we present as the electronic relaxation processes after optical excitation. Strong optical excitation heats the free electrons to a high temperature, which not only induces oscillation of the free electrons, but also produces strong intraband transitions within the conduction band. Here, we assume that the excitation photon energy is lower than the threshold for interband transitions. As shown in Figure 1b, gold has a threshold for the 5d‐to‐6sp interband transition around 500 nm (2.48 eV). Strong optical excitation may induce large‐scale electronic transitions within the conduction band, as highlighted by the upward arrow in magenta in Figure 1b, which is assigned as intraband transitions. Such a strong excitation leads to transient “depletion” of the conduction band just below the Fermi level, being equivalent to a “transient lowering” of the Fermi level, as highlighted by a downward arrow in Figure 1b. Consequently, the threshold for interband transition is lowered slightly, as highlighted by the leftward arrow. Thus, the photon energy allowed for interband transitions is reduced, so that a transient absorption spectrum is generally observed at the interface between the intra‐ and inter‐band edges.

The hot electrons (Δ*N*) excited through intraband transition will relax back to their original states through complex transition channels, which may be roughly characterized by $\frac{d\Delta N(t)}{dt}=\;P(t)\Delta N(t)-\frac{\Delta N(t)}{\tau_{h}}$, where *P*(*t*) is the pump rate defined by the temporal intensity distribution of the pump pulse and τ_h_ is the lifetime of the hot electrons for transferring back below the Fermi surface. Meanwhile, another portion of the electrons is driven by the excitation pulse to oscillate plasmonically near the Fermi surface. After coherent interaction with the excitation optical electric field, these plasmonic electrons relax mainly through energy transfer to the phonons, resulting in the heating of the lattices and consequently linear expansion of them, as depicted schematically in Figure 1c. It needs to be noted that this portion of the electrons includes a contribution from the relaxing hot electrons. The enlargement of the lattice constant (*a*) can be characterized by the change of *a* by a factor of (1+*Δ*%). Although optical heating induces very small expansion of the lattices, the bandstructures are very sensitive to such a modulation. Figure 1d shows the calculated density of states (DOS) for gold as a function of the relative energy with respect to the Fermi level as the linear expansion of the lattices is increased from 0% to 2%. As indicated by arrows in Figure 1d, the bandgap is reduced due to such a lattice expansion, implying redshift of the threshold for interband transitions. Meanwhile, the Fermi level shifts linearly to the red, as shown in the inset of Figure 1d by the Fermi energy (*E*_F_) as a function of the lattice expansion ratio (ER). However, we need to be aware that this shift of the Fermi level is not a direct result of the optical heating of the electrons, instead, it results from the enlargement of the lattice constant due to thermal expansion. For a visible spectral band (1.77–3 eV), we present an enlarged view of the calculated bandstructure in Figure 1e, corresponding to the circled region by a dashed ellipse in Figure 1d. Even for a linear expansion ratio as small as 0.1%, we will expect a redshift of the lower edge of the excitation band by roughly 33 meV. As highlighted by the dashed arrows in Figure 1e, at a photon energy of 2.48 eV and ER = 2%, the redshift can be as large as Δ*E* = 194 meV.

Above analysis suggests that optical excitation modulated the electronic bandstructures of gold in two stages. In the first, optical excitation induces strong intraband transitions and a transient depletion of the conduction band electrons below the Fermi level, which lowers the threshold photon energy for interband transition and leads to redshift of the corresponding absorption spectrum. In the second stage, the hot electrons release their energy to the lattices through electron–phonon interaction, resulting in the heating and linear expansion of the lattices. Lattice expansion lowers the edges of the excitation band and induces redshift of the absorption spectrum. Thus, lattice expansion in the later stage modulates the electronic bandstructure in the same direction as the intraband electronic transition in the early stage. These processes account for the two‐stage evolution dynamics in the transient spectroscopic response of the gold nano film (AuFilm) and gold nanoparticles (AuNPs) on the bandedge.

### 2.2 Thermal Excitation and Continuous‐Wave Optical Excitation

**Figure 2:**
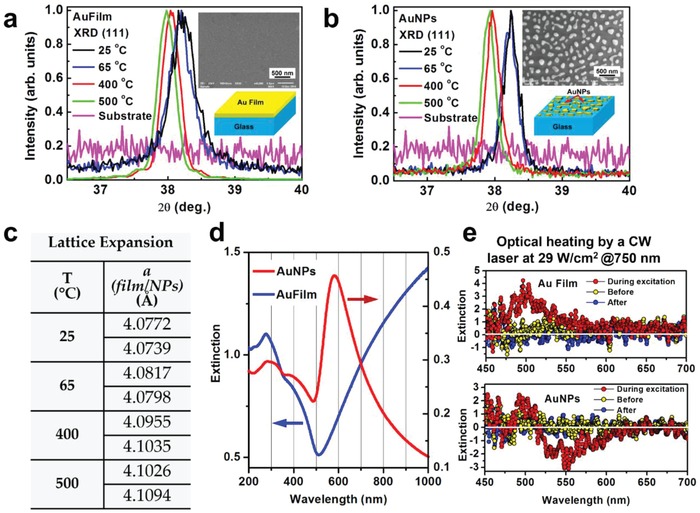
a,b) X‐ray diffraction measurements on AuFilm and AuNPs, respectively, at different temperatures with comparison to that on a pure glass substrate. Insets: SEM images of the gold nanostructures and the structural illustration of the samples. c) The lattice constants of the gold nanostructures calculated from the XRD measurements in (a) and (b). d) Optical extinction measurements on the samples in the insets of (a) and (b). e) Optical extinction measurements on the samples in (a) and (b) when they are heated optically by a CW laser beam at 750 nm with a power intensity of 29 W cm^−2^. The transmission spectrum without optical excitation was used as the blank for the calculation of the optical extinction spectrum. Comparison between measurement before, after, and during the laser excitation is supplied by the yellow, blue, and red filled circles.

To characterize the thermal expansion effects on the nanostructured gold, we carried out X‐ray diffraction (XRD) measurements on AuFilm and AuNPs at different temperatures, as shown in **Figure** **2**a,b, respectively. The AuFilm was deposited on a glass substrate by thermal evaporation, which has a thickness of 50 nm. The AuNPs were produced by annealing colloidal gold nanoparticles at 400 °C. The chemically synthesized gold nanoparticles were dissolved in xylene with a concentration of 100 mg mL^−1^ and were spin‐coated onto a glass substrate at a speed of 2000 rpm for 30 s to produce a homogeneous layer before the annealing process. The upper panels of the insets of Figure 2a,b present the scanning electron microscope (SEM) images of the AuFilm and AuNPs, respectively, and the lower show schematically the construction of the samples. The XRD patterns were captured at different temperatures using an Ultima IV X‐ray diffraction system with Cu Kα radiation.

The XRD measurements on pure glass substrates, as shown by the curves in magenta are included in Figure 2a,b for comparison, which rule out the possibility of modulation by the glass substrate and verify that the XRD peaks are solely from the X‐ray diffraction by gold. As shown in Figure 2a, when the temperature was increased from 25 °C (black) to 65 °C (blue), 400 °C (red), and 500 °C (green), the XRD peak at the (111) crystal plane for the AuFilm shifted from about 38.24° to 38.19°, 38.05°, and 37.97°, implying an obvious enlargement of the lattice constant by thermal expansion. The corresponding lattice constants were calculated to be 4.0772, 4.0817, 4.0955, and 4.1026 Å, respectively, as listed in Figure 2c. Similar effects can also be observed for the AuNPs, as shown in Figure 2b, where the XRD patterns were acquired at a same group of temperatures and at the same (111) crystal plane. The XRD peak angles were measured to be 38.24°, 38.21°, 37.97°, and 37.90°, thus, the corresponding lattice constants for AuNPs were calculated to be 4.0739, 4.0798, 4.1035, and 4.1094 Å, respectively. Comparison between the enlarged lattice constants due to thermal expansion with increasing the temperature, as shown in Figure 2c, indicates that the AuNPs exhibit slightly larger thermal expansion ratio than the AuFilm, meanwhile, the XRD pattern is narrower for the AuNPs than for the AuFilm, which can easily be inferred by a comparison between Figure 2a,b. We can also conclude from the experimental results in Figure 2a–c that even a temperature increase of 40 °C (from 25 to 65 °C) may induce a linear expansion ratio of larger than 0.1%. According to the calculation results in Figure 1e, such an expansion can already results in a bandedge shift of 33 meV, which is obviously observable in the optical spectroscopic response.

It needs to be noted that in the thermal expansion experiment, the sample was heated on an enclosure hotplate installed with the XRD equipment. To rule out the possibility of the substrate‐expansion induced lattice expansion, we performed continuous optical heating of the gold nanostructures using a laser beam at 750 nm, at which the glass substrate has nearly no absorption of light and was not heated by the laser irradiation. Before the laser‐heating experiment, we first measured the steady‐state optical extinction spectra of the AuFilm (blue) and AuNPs (red), as shown in Figure 2d. Both spectra show a threshold wavelength for interband transitions at about 500 nm (2.48 eV), where we can observe a deep dip for both spectra. Localized surface plasmon of the AuNPs can be observed as a featured spectrum with a peak at 580 nm and a bandwidth of 144 nm at full width at half‐maximum (FWHM). When heating the AuFilm using the 750 nm laser beam with an intensity of 29 W cm^−2^, we observe an obvious spectral feature peaked at 500 nm and extending from about 450 to 625 nm, as shown by the red filled circles in the upper panel of Figure 2e. The yellow and blue filled circles are the measurements before and after laser irradiation, respectively, where no modification can be observed, implying that the AuFilm sample was not damaged by the continuous laser irradiation and the spectral feature by the red circles is truly the spectroscopic response of the AuFilm to the optical heating. The lower panel of Figure 2e shows the optical spectroscopic response of the AuNPs to the optical heating by laser irradiation at 750 nm with the same intensity as that used for the AuFilm. A similar spectral peak at 500 nm is observed; however, an additional dip at about 550 nm makes the spectroscopic response completely different at longer wavelengths than 520 nm from that for the AuFilm. However, the negative spectral feature also extends to as long as 625 nm. Therefore, for the positive spectral features, the AuFilm and the AuNPs exhibit quite similar response, which can be assigned as the optical excitation induced transient bandedge shift due to heating and expansion of the lattices. These processes are clearly not dependent on the size and shape of the gold nanostructures, i.e., they are intrinsic of gold, which will be verified further with more solid evidence by the transient spectroscopic response in Section 2.3.2. However, for the spectral difference at wavelengths longer than 500 nm, we have to consider localized surface plasmons (LSPs) of the AuNPs, which agrees well with the fact that 500 nm is the lower edge for interband transitions and the higher edge for the plasmon band. Redshift of the threshold for interband transitions weakened the original mechanisms for LSP and reduced the optical extinction by plasmonic oscillation of the free electrons. Considering that intraband transitions and plasmonic oscillations are both ultrafast processes, femtosecond spectroscopy is employed to disclose the true mechanisms for the electronic and plasmonic processes in the AuNPs and the AuFilm.

### 2.3 Ultrafast Optical Excitation

#### 2.3.1 Two‐Temperature Modeling

Theoretical analysis based on the two‐temperature model is first carried out to understand how the 50 nm AuFilm responds to the excitation by ultrashort laser pulses. The two‐temperature model for describing the dynamics of electronic (*T*_e_) and phononic (*T*_l_) temperatures under optical pulse excitation can be simplified and characterized as follows

(1) $$C_{e}\;\frac{dT_{e}\left(t\right)}{dt}=\;-g\left[T_{e}\left(t\right)-T_{l}\left(t\right)\right]+S\left(t\right)$$

(2) $$C_{l}\;\frac{dT_{l}\left(t\right)}{dt}=\;g\left[T_{e}\left(t\right)-T_{l}\left(t\right)\right]$$

where *C*_e_ and *C*_l_ are the specific heat capacities of electrons and lattices, respectively, *g* is the electron–phonon coupling constant as defined in ref. 42 and *S*(*t*) denotes the optical excitation intensity. For a pump pulse fluence *P*, we define $S\;(t)=\;A\frac{\alpha P}{\tau_{P}}e^{-4\ln2{\left(\frac{t}{\tau_{P}}\right)}^{2}}$, where *A* is a constant, α is the absorption coefficient, and τ_P_ is the pulse length at FWHM.

**Figure 3:**
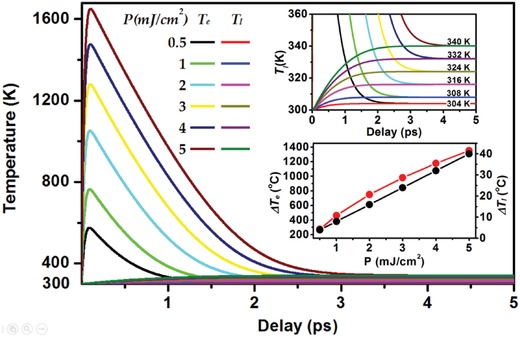
Calculated temperature of hot electrons and lattices after excitation by optical pulses at about 800 nm with a pulse length of 150 fs and a pulse energy fluence (*P*) ranging from 0.5 to 5 mJ cm^−2^. Inset: Enlarged view of the dynamics for a delay time range of 0–5 ps and a temperature range of 300–360 K (upper panel) and the temperature increase of the electrons (Δ*T*_e_) and lattices (Δ*T*_l_) as a function of pump fluence (lower panel).

**Figure** **3** shows the calculated evolution dynamics of *T*_e_ and *T*_l_ after excitation by 150 fs laser pulses at 800 nm with a pump fluence varied from 0.5 to 5 mJ cm^−2^. In the calculation, we employed the following parameters: *C*_e_ = σ_e_ *T*_e_ with σ_e_ = 68 J m^−3^ K^−2^, *C*_l_ = 2.44 × 10^6^ J m^−3^ K^−1^, *g* = 7 × 10^16^ W m^−3^ K^−1^.

As the pump fluence was increased from 0.5 to 1, 2, 3, 4, and 5 mJ cm^−2^, the peak electron temperature reaches 575, 763, 1050, 1280, 1475, and 1650 K, respectively. Meanwhile, the lattice temperature increases gradually with energy transferred from electrons to the lattices. The corresponding equilibrium process takes 2.5, 2.8, 3.3, 3.6, 3.8, and 4.3 ps, respectively, implying that more time is required for hotter electrons to release their energies to the lattices. This is more clearly seen in the inset of Figure 3 by the upper panel, where the lattice temperature is increased to 304, 308, 316, 324, 332, and 340 K from 293 K. The corresponding temperature increase is 11, 15, 23, 31, 39, and 47 °C, respectively. The lower panel of the inset of Figure 3 plots the neat temperature increase of the electrons (Δ*T*_e_) and lattices (Δ*T*_l_) as a function of the pump fluence. Although the Δ*T*_e_–*P* curve exhibits some nonlinearity, Δ*T*_l_ varies nearly linearly with *P*, implying that the heating of the lattice is proportional to the excitation pulse energy. The nonlinearity in the Δ*T*_e_–*P* relationship can be explained by the extended decay dynamics of the electron‐involving process. On such bases, the calculation results supply evidence that the heating by femtosecond optical excitation is strong enough to induce lattice expansion and consequent redshift of the Fermi level, as demonstrated in Figure 2.

#### 2.3.2 Transient Spectroscopic Response on Femtosecond Pulse Excitation

In femtosecond transient absorption (TA) spectroscopic investigation, a Ti:sapphire amplifier supplied laser pulses at 800 nm with a pulse length of about 150 fs, a repetition rate of 1 kHz, and a maximum pulse energy of 1 mJ. A portion of the 800 nm pulse was sent to heavy water in a cuvette with a thickness of 3 mm to produce the supercontinuum pulses, which were used as the probe. The rest of the 800 nm pulse was used as the pump, which has a maximum pulse fluence of about 5 mJ cm^−2^. According to the theoretical results in Figure 3, such a pump fluence may lead to a temperature increase of 47 °C for the lattices. If we look back at Figure 2, such a temperature increase corresponds to an ER as large as 0.15%. The corresponding bandedge shift can be more than 33 meV, as can be evaluated from Figure 1e, which is large enough for modifying the optical absorption spectrum. However, the low photon energy (≈1.55 eV) of the pump pulses implies no direct excitation of interband transitions. AuFilm and AuNPs characterized in Figure 2a,b, respectively, were taken as the samples for investigation.

**Figure 4:**
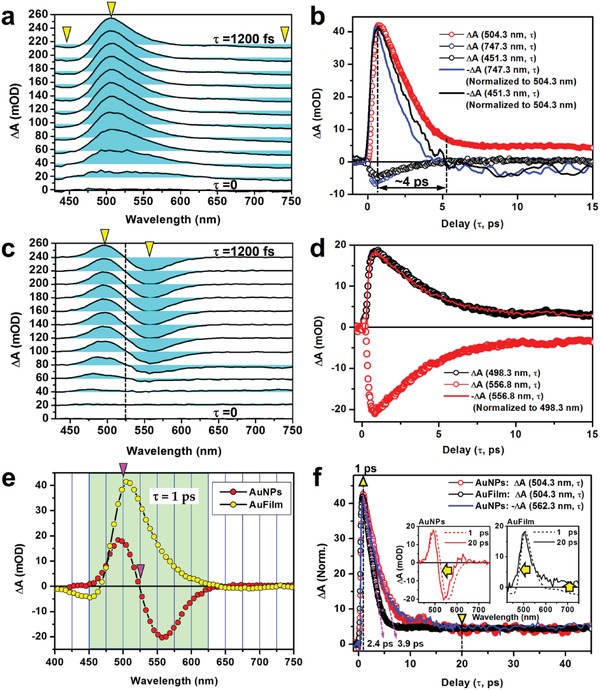
Transient absorption (TA) measurements on the 50 nm gold film (AuFilm) and the gold nanoparticle matrix (AuNPs). a) TA spectra measured on the AuFilm for a time delay of 0–1200 fs. b) TA dynamics measurements on the AuFilm at 451.3 nm (black circles), 504.3 nm (red circles), and 747.3 nm (blue circles). The TA dynamics at 451.3 (black curve) and 747.3 nm (blue curve) are negated and normalized to that at 504.3 nm for comparison. c) TA spectra measured on the AuNPs for a time delay of 0–1200 fs. d) TA dynamics measured on the AuNPs at 498.3 nm (black circles) and 556.8 nm (red circles). The TA dynamics at 556.8 nm (red curve) is negated and normalized to that at 498.3 nm for comparison. e) Comparison between the TA spectra measured on AuNPs and AuFilm at a delay of τ = 1 ps. f) Comparison between TA dynamics measured on AuNPs (red circles) and AuFilm (black circles) at 504. 3 nm, and on AuNPs at 562.3 nm after being negated and normalized (blue curve). Insets: comparison between the TA spectra at a delay of 1 and 20 ps for the AuNPs (left) and AuFilm (right).

**Figure** **4**a shows the TA spectra measured on AuFilm for delays of 0–1200 fs in steps of 100 fs. A most important feature can be observed as a positive TA spectrum peaked at about 505 nm, which extends from about 450 to 625 nm and is accompanied by small negative features on both sides, as highlighted by yellow triangles. The positive spectrum reaches its maximum at about τ = 630 fs, as can be measured by the TA dynamics in Figure 4b, where we plot the measurements at 451.3, 504.3, and 747.3 nm by black, red, and blue circles, respectively. Locations of these three wavelengths are also highlighted by downward triangles in Figure 4a. For better comparison, we have taken the opposite of the negative dynamics at 451.3 and 747.3 nm and normalized them to the positive at 504.3 nm, as shown by the plots of black and blue curves, respectively. We assign the strong positive TA spectrum as the enhanced transient absorption on the boundary between interband and plasmon‐band processes, which was induced by the transient “depletion” of the conduction band within a “top layer” just below the Fermi level and the consequent transient “lowering” of the threshold for interband transition, as depicted in Figure 1b. The original boundary between inter‐ and intra‐band transitions was pushed to the red. Thus, the transient interband transition becomes allowed for the original upper edge of the plasmonic band. Relaxation of this transient bandedge shift takes about 4 ps before electrons release their energy to the lattices, as shown by the red circles and scaled by the double arrow in Figure 4b.

The lattice expansion induced by the heat transfer from the hot electrons leads to lowering of the Fermi level, as has been demonstrated in Figure 1d,e. Similar to the effects due to transient electronic band depletion, the lowering of the Fermi level also lowers the threshold for interband transitions and results in optical absorption in a redshifted spectral band. This lattice‐expansion‐induced optical absorption takes a much longer time, as can be inferred from Figure 3 and observed in Figure 4b at τ > 5 ps.

The negative feature below 460 nm is partially due to the bleaching of the interband transitions through two‐photon excitation by the pump pulses and partially due to the bandstructure modulation by the strong optical excitation. In contrast, the negative band on the right side of the positive spectrum at wavelength longer than 625 nm can be explained by the bleaching of the absorption, scattering, and reflection of light by the mass free electrons in AuFilm. However, both of these negative features become positive at τ > 5 ps, verifying that lattice expansion induced bandstructure modulation and the consequently enhanced optical absorption play consistently a dominant role over the whole spectral band. However, it takes a few picoseconds to reach the equilibrium for the electron–phonon interaction, which counteracts the negative TA signal. This explains the relatively faster dynamics at 451.3 and 747.3 nm than that at 504.3 nm, as shown in Figure 4b, where the falling edges of the blue and black curves were cut steeper. It needs to be noted further that both 451.3 and 747.3 nm are located on the edges of the band structures of gold, according to Figure 1d, implying that the dynamics there is more sensitive to the electronic bandedge shift.

Although the steady‐state optical extinction by LSP of the AuNPs extends from 500 to longer than 800 nm, as shown in Figure 2d, the TA spectrum corresponding to reduced optical extinction by LSP covers a much narrower band from about 525 to 625 nm, which is defined by the overlap of the LSP spectrum with the bandstructure modulation by optical excitation as confirmed by Figure 4c, where we plot the TA spectra measured on the AuNPs for delays of 0–1200 fs in steps of 100 fs. In Figure 4d, we show the TA dynamics at 498.3 nm (black circles) and 556.8 nm (red circles), corresponding to the peak and dip wavelengths in Figure 4c, respectively, as highlighted by the yellow triangles. We negated the TA dynamic curve at 556.8 nm and normalized it to the positive peak at 498.3 nm, which is plotted by the red curve in Figure 4d. Clearly, the red curve overlaps precisely the black circles, implying that the same mechanism is responsible for the positive and negative TA dynamics peaked at 498.3 and 556.8 nm, respectively. This is a further verification of the bandedge‐shift mechanisms. In Figure 4e, we plot the TA spectra at a delay of 1 ps for the AuFilm and the AuNPs. Clearly, the spectral features for AuFilm and AuNPs are overlapped in the same range from about 450 to 625 nm, as highlighted by the light‐green background, implying a similar mechanism supporting the spectroscopic modulation. At such a delay, the dominant mechanism is the transient depletion of the electronic conduction band below the Fermi level.

In Figure 4f, we plot the TA dynamics measured on the AuFilm (black circles) and AuNPs (red circles) at a same wavelength of 504.3 nm, as well as the inversed TA dynamics for the AuNPs at 562.3 nm (blue curve). Clearly, the AuNPs have the same lifetime for the decay dynamics at 504.3 and 563.3 nm. These three dynamics were normalized to the peak at a delay of τ = 1 ps. All dynamic curves evolve into a fast and a slow stage. The fast one corresponds to the large‐amount excitation of hot electrons and strong intraband transition induced transient depletion of the local conduction band, which is an “equivalent” transient lowering of the Fermi level. These hot electrons release their energy to the lattices and come back to their original states after a few picoseconds. The heating induces expansion of the lattices in the AuFilm and AuNPs, resulting in a “true” lowering of the Fermi level and continuing the modulation on the absorption spectrum. This process relaxes over a much longer time scale, constituting the slow stage of the TA dynamics in Figure 4f.

A lifetime of about 2.4 ps for the AuFilm and 3.9 ps for the AuNPs can be resolved from the fittings to the fast dynamics in the first stage, as highlighted by the dashed lines in magenta in Figure 4f. The much slower decay dynamics of the AuNPs than the AuFilm can be understood by considering the much smaller total volume and much larger total surface for optical absorption of the AuNPs than the AuFilm. The electrons in AuNPs are more strongly excited but more strongly limited in releasing their energy. Therefore, it takes more time for the electrons to relax through heating the lattices and come back to their original states.

The insets of Figure 4f supply a comparison between the TA spectra at 1 and 20 ps for the AuNPs (left) and AuFilm (right), which correspond to the fast and slow decay dynamics, respectively, as indicated by the yellow triangles. These spectra have been normalized for better comparison. Apparently, they basically keep their shapes for the evolution from the fast to the slow stage; however, there is an obvious blueshift of the TA spectrum, as indicated by the leftward arrows in both insets, implying that: 1) optical excitation of the Au nanostructures has induced a redshift of the TA spectrum and the recovery of the system exhibits a blueshift; (2) the electronic band depletion effect modulates the transient spectroscopic response more strongly than the lattice expansion effect; 3) the electronic band depletion and the lattice expansion induced similar effects and pushed the bandedge shift in the same direction. All of these mechanisms favor redshift of the threshold for interband transitions and push the plasmonic band further to longer wavelengths, thus explaining consistently the transient spectroscopic response in Figure 4.

Based on above analysis, we may look back at the negative to positive transition features highlighted by the upward arrow in the right inset of Figure 4f. Due to the strong transient depletion through intraband transitions, the plasmonic absorption over the whole spectrum was bleached, explaining the negative features in the TA spectrum at a delay of τ = 1 ps. However, the depletion effect has a much faster dynamics than the lattice heating and the induced expansion effect. Therefore, at τ = 20 ps the depleted electronic band recovers and the lattice‐expansion‐induced Fermi‐level shift becomes dominant, which is observed as a positive TA spectrum. Such a thermal effect takes much longer time to relax.

We need to stress that the TA response in Figure 4 cannot be explained by the generally assigned plasmonic e–e, e–p, and p–p scattering processes. Both e–e and e–p scattering processes are only involved in the plasmonic band and result in the redshift of the resonance spectrum, so that “left negative” with “right positive” features should be observed in the TA spectrum plotted with a wavelength horizontal axis. However, these characteristic features cannot be resolved from any of experimental observations. Thus, the transient “electronic depletion” and “lattice expansion” effects constitute the true physical mechanisms for understanding the spectroscopic modulations on the bandedge between interband transitions and plasmonic electron oscillations.

The transient spectroscopic performance in Figure 4 verifies further that the optical excitation induced “Fermi‐level shift” by transient electronic depletion and lattice expansion is intrinsic with gold, which is thus independent of the size and shape of the nanostructures. The conclusive evidence was not only supplied by the comparison of the TA spectroscopic response between AuFilm and AuNPs in Figure 4, but also verified by experimental results on gold nanowires (AuNWs) in the supporting information. In Figure S1 (Supporting Information), we compare the TA spectrum and dynamics measured on the AuNWs with those on the AuNPs in Figure 4. The SEM image of the AuNW grating is shown in Figure S1a (Supporting Information). As shown in Figure S1b (Supporting Information), the TA spectra at an equal time delay exhibit quite similar features for AuNWs and AuNPs, as indicated by downward triangles. Meanwhile, the dynamic curves at 498.3 nm in Figure S1c (Supporting Information) show nearly the same lifetime of the TA decay process for AuNWs and AuNPs.

## 3 Conclusions

We discovered that “transient depletion of the electronic conduction band” and “transient lattice expansion” induced Fermi‐level shift has been the true mechanism for the strong spectroscopic modulation on the boundary between interband transition and plasmonic electron oscillation by optical excitation of the metallic nanostructures. These bandedge processes correspond to the fast and the slow evolution dynamics that are conventionally assigned as the electron–phonon and phonon–phonon interaction processes, respectively. Transient spectroscopic investigations, temperature‐changing XRD measurements, and theoretical analysis using first‐principles calculations and two‐temperature modeling supply multifold evidence and consistent supports to the conclusions. The revealed mechanisms give new insights into the plasmonic physics and are thus important for the applications of plasmonic micro‐ and nanostructures in optoelectronic devices. More specifically, these mechanisms can be utilized or exploited in the development of optical switching devices and optical logic circuits. In particular, the transient adjustment of the Fermi level via optical excitation also supplies new approach for the design of optoelectronic devices with metal–semiconductor interfaces, which is important for photoelectric detection.

## Conflict of Interest

The authors declare no conflict of interest.

## Supporting information

Supporting Information
