# Supplementary material for: Transient Electronic Depletion and Lattice Expansion Induced Ultrafast Bandedge Plasmons
Source: Adv Sci (Weinh). 2019 Nov 27;7(2):1902408. doi: 10.1002/advs.201902408 (PMC6974950; doi:10.1002/advs.201902408)
Supplement: Supplementary file 1 — Supporting Information [file ADVS-7-1902408-s001.pdf]

Copyright WILEY-VCH Verlag GmbH & Co. KGaA, 69469 Weinheim, Germany, 2019.

ADVANCED SCIENCE
Open Access

Supporting Information

for *Adv. Sci.,* DOI: 10.1002/advs.201902408

Transient Electronic Depletion and Lattice Expansion Induced Ultrafast Bandedge Plasmons

Xinping Zhang, Meng Wang, Fawei Tang, Huanzhen Zhang, Yulan Fu, Dong Liu, and Xiaoyan Song**

Copyright WILEY-VCH Verlag GmbH & Co. KGaA, 69469 Weinheim, Germany, 2019.

Supporting Information

Transient electronic depletion and lattice expansion induced ultrafast bandedge plasmons

Xinping Zhang, Meng Wang, Fawei Tang, Huanzhen Zhang, Yulan Fu, Dong Liu, and Xiaoyan Song**

Emails: zhangxinping@bjut.edu.cn; xysong@bjut.edu.cn

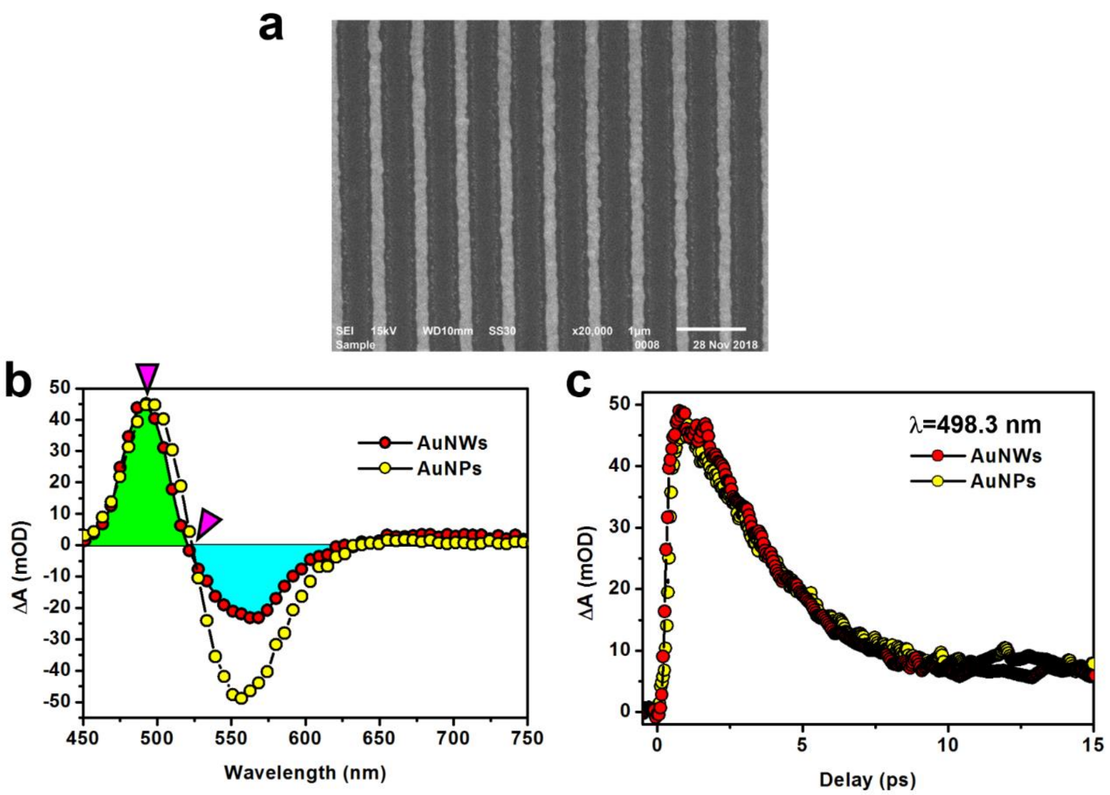

Fig. S1 (a) SEM image of a gold nanowire (AuNW) grating fabricated on a glass substrate. (b) Transient absorption (TA) spectra measured on AuNWs (solid red circles) and AuNPS (solid yellow circles) at a delay of 1 ps in Fig. 4(e), showing consistent typical spectral positions, as marked by triangles. The two TA spectra were normalized at about 500 nm. (c) Comparison between the TA dynamics measured on AuNWs (solid red circles) and AuNPs (solid yellow circles). Two dynamics curves have been normalized at the peak intensity.
